# Posterior Electrical Axis Deviation Is Associated With Future Development of Left Bundle Branch Block: A UK Biobank Study

**DOI:** 10.1016/j.jacadv.2026.102842

**Published:** 2026-06-17

**Authors:** Mohammad Kayyali, Ana Mincholé, Martin Bishop, Hassan Zaidi, Shuang Qian, Steven Niederer, Rachel Bastiaenen, Chris Miles, Pablo Lamata, John Whitaker

**Affiliations:** a School of Biomedical Engineering and Imaging Sciences, King’s College London, London, United Kingdom; b BSICoS, IIS Aragón, University of Zaragoza, Zaragoza, Spain; c National Heart and Lung Institute, Imperial College London, London, United Kingdom; d The Alan Turing Institute, London, United Kingdom; e Guy's and St Thomas' NHS Foundation Trust, London, United Kingdom

**Keywords:** electrical axis, electrocardiogram, left bundle branch block, UK Biobank

## Abstract

**Background:**

Left bundle branch block (LBBB) describes a specific cardiac conduction abnormality which may be a cause, consequence, or exacerbator of cardiovascular dysfunction and events. Electrocardiogram-derived definitions, which depend heavily on QRS duration (QRSd), are imperfect and continue to evolve, with limited data on predicting LBBB development.

**Objectives:**

This study aimed to determine whether changes in electrical axis identify patients at risk of future development of LBBB.

**Methods:**

Retrospective data from 35,749 UK Biobank participants were analyzed, excluding those with overt cardiovascular disease. The primary endpoint was LBBB development. Associations with axis metrics (computed from baseline cardiac magnetic resonance imaging and 12-lead electrocardiogram) were investigated using Kaplan-Meier analysis and Cox proportional hazards models adjusted for age, sex, hypertension, left ventricular ejection fraction and QRSd.

**Results:**

The cohort (age 63.4 ± 7.6 years, 45% male, QRSd: 87.4 ± 12.8 ms) was followed for a median of 6 years. Compared with the event-free population (N = 35,688), those who developed LBBB (N = 41) were older (69.2 vs 64.0 years; *P* < 0.001), more likely to have hypertension (31.7% vs 11.6%; *P* < 0.001), and had a significantly lower (more posterior) electrical axis (*φ*_Electrical_: 69.1° vs 80.3°; *P* = 0.01). In multivariable analysis, lower *φ*_Electrical_ (HR: 0.73; 95% CI: 0.56-0.94; *P* = 0.014) was significantly associated with incident LBBB. Individuals with both high QRSd and low *φ*_Electrical_ had a four-fold increased risk (HR: 4.09; 95% CI: 1.84-8.99; *P* < 0.001).

**Conclusions:**

Posterior deviation of the transverse electrical axis complements QRSd in identifying individuals at risk of developing LBBB. These findings suggest that the electrical axis is capturing early conduction disease and may contribute to risk stratification of future development of LBBB.

Left bundle branch (LBB) block (LBBB) is a cardiac conduction system disorder that describes failure of conduction within the LBB of the specialized conduction system. This results in characteristic features on 12-lead electrocardiogram (ECG) from which the condition is most commonly diagnosed. The presence of LBBB is considered an adverse cardiac feature, which can lead to progressive left ventricular (LV) dysfunction and heart failure (HF) and is associated with increased cardiovascular mortality.^1^ First described by Eppinger et al^2^ in 1909, the ECG features typical of LBBB result from failure or significant slowing of the electrical impulse at some point within the left-sided conduction system.^1^ This results in antegrade myocardial activation exclusively via the right bundle branch (RBB) of the conduction system which terminates in the free wall of the right ventricle. As a result of failure to engage the left-sided conduction system, LV electrical activation spreads by transmyocardial conduction from tissue activated by the RBB. Slow LV activation may result in electrical and mechanical dyssynchrony, manifesting as a widened QRS complex and the characteristic ECG features of LBBB.

Other conduction abnormalities which significantly delay LV activation may also produce a surface QRS transcription that mimics true LBBB.^3^ As such, the ECG appearances of LBBB may reflect true conduction block in the left bundle or other pathologies, including conduction delay within the fascicles or Purkinje fibers, or more diffuse myocardial alterations, such as redistribution of gap junction proteins (eg connexin 43). As well as mimicking LBBB, these pathologies may also produce a spectrum of abnormalities and LBBB-like patterns not fully captured by the standard ECG criteria.^1^^,^^4^^,^^5^

In practice, a diagnosis of LBBB is based predominantly on QRS duration (QRSd) and morphological features. However, there is substantial variation and frequent changes to guideline definitions used to diagnoses LBBB from ECG. This reflects an attempt to optimize the sensitivity and specificity with which a diagnosis of LBBB may be made from ECG. In 2009, the American Heart Association , American College of Cardiology Foundation, and Heart Rhythm Society have established consensus criteria (updated in 2018) centered around QRSd ≥120 ms, in addition to basic morphological features such as a broad notching or slurring of the R wave and the absence of Q wave in certain leads.^5^^,^^6^ Building on these, Strauss et al.^7^ proposed more stringent criteria, including higher sex-dependant QRSd thresholds and additional morphological requirements, with the aim of better identifying patients most likely to benefit from cardiac resynchronization therapy (CRT), for which true LBBB is considered a key indicator of likely response. Most recently, the European Society of Cardiology (ESC) 2021 guidelines introduced stricter and more detailed ECG criteria, emphasizing QRSd and specific features of QRS morphology (QRSd >120 ms, notching or slurring in the middle third of the QRS complex in multiple leads and delayed R wave peak in V5–V6, T-wave asymmetry and polarity, and ST changes).^8^ Although these changes are intended to improve diagnostic specificity, they are at the cost of reduced sensitivity and therefore potentially risk excluding patients who might benefit from CRT.^9^

The increasing focus on ECG morphology beyond duration alone highlights the role of the specific alterations in conduction pattern. Given the limitations of traditional 12-lead ECG in fully capturing the complex 3-dimensional (3D) relationship between myocardial architecture, cardiac geometry, and electrical propagation, cardiac electroanatomical axes have emerged as quantitative metrics that can provide distinct information about this relationship beyond what is captured by conventional ECG parameters.^10^^,^^11^

There are limited ECG features that have been validated as predictive of the future development of LBBB; however, abnormalities in the performance of the LBB are also well recognized, including for example failure of conduction in one of the limbs of the LBB, such as left anterior fascicular block (LAFB).^12^ Although studies such as the SPRINT trial have identified general risk factors for LV conduction disease,^13^ there remains a notable absence of validated ECG phenomena that represent early conduction system abnormalities preceding LBBB. A strategy to identify patients at risk of developing LBBB would be of value for the identification of patients who may be at a higher risk of conduction system disease progression and the adverse consequences of development of LBBB in the future.

In this study, we aimed to investigate the utility of electrical and anatomical axis metrics in adjunct to QRSd for assessing the risk of LBBB development. The cohort included 35,749 UK Biobank participants recruited retrospectively and free from baseline conduction abnormalities, ischemic, or structural heart disease, and assessed associations using Kaplan-Meier and Cox proportional hazards analyses.

## Methods

### Study design and population

This study used data from the UK Biobank, a large biomedical database of over 500,000 participants aged 40 to 69 years.^14^ Electronic health records were linked, allowing access to follow-up information including hospital inpatient records and death registries. The UK Biobank received ethical approval from the North-West Multicenter Research Ethics Committee (REC reference: 11/NW/03,820), and written informed consent was obtained from all participants before enrollment in accordance with the Declaration of Helsinki.

**Figure 1:**
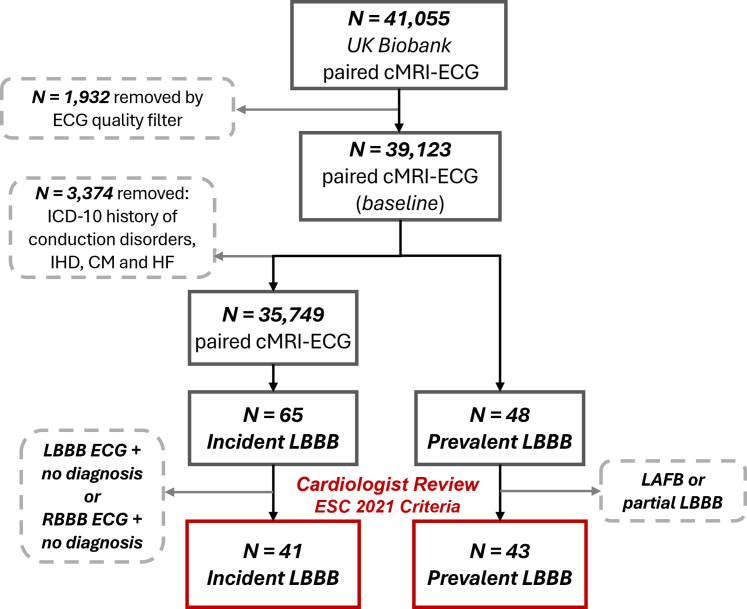
Study Population Flowchart Showing the Final Study Cohort CM = cardiomyopathy; cMRI= cardiac magnetic resonance imaging; ECG = electrocardiogram; ESC = European Society of Cardiology; HF = heart failure; ICD-10: International Classification of Diseases, 10th Revision; IHD = ischemic heart disease; LAFB = Left anterior fascicular block; LBBB = left bundle branch block; RBBB = Right bundle branch block. LBBB ECG with no diagnosis was added to the prevalent LBBB group.

Invited participants underwent a single imaging visit including paired cardiac magnetic resonance imaging (cMRI)-ECG acquisition. These investigations were performed for research purposes rather than clinical indications. From 41,055 participants with paired cMRI-ECG data (Figure 1), participants were excluded if they had any of the following baseline conditions: conduction abnormalities (including atrioventricular block, fascicular block, bundle branch block, or nonspecific intraventricular conduction delay), arrhythmias (including atrial fibrillation/flutter, supraventricular or ventricular arrhythmias), ischemic heart disease, cardiomyopathy (CM), and HF. This was defined by codes from the International Classification of Diseases-10th Revision (ICD-10) of the linked hospital records. Participants were classified as having hypertension based on ICD-10 codes for primary hypertension (I10).

A cardiac electrophysiologist manually reviewed the baseline ECGs of all participants flagged with a pre-existing or subsequent LBBB diagnosis to ensure the diagnostic accuracy. Participants with baseline fascicular block were excluded, and manual ECG review confirmed no fascicular block morphology in the remaining cohort. Using the stricter 2021 ESC criteria,^8^ prevalent LBBB participants whose baseline ECG did not meet the criteria were excluded, and those originally coded as incident LBBB but found to have LBBB morphology at baseline were reclassified as prevalent (Supplemental Table 1). Baseline was defined as the time of ECG and cMRI acquisition; prevalent refers to LBBB diagnosis at or before baseline, whereas incident refers to new LBBB that developed after baseline.

### Outcome definition

The primary outcome was a diagnosis of LBBB during follow-up, defined using ICD-10 codes from linked hospital records. UK Biobank’s participant records are continuously updated with diagnostic codes and corresponding dates. The follow-up began at the baseline assessment and ended at the earliest of: 1) first LBBB diagnosis; 2) death; or 3) end of follow-up (the latest available date of record update).

### Data acquisition

All cMRIs were obtained using UK Biobank’s standardized, ECG-gated protocol; detailed scanner parameters and acquisition settings have been previously reported.^15^ An automated process generated 3D surface reconstructions of the biventricular anatomy from end-diastolic frames, as previously described.^16^^,^^17^ Resting digital 12-lead ECGs were stored using the GE CASE/Cardiosoft v6 system. The median ECG from a 10-second recording was converted into vectorcardiogram using the Kors transformation,^18^ capturing how the cardiac dipole changes both in magnitude and orientation throughout the cardiac cycle. Further details are in Supplemental Methods.

### Metric computation

**Figure 2:**
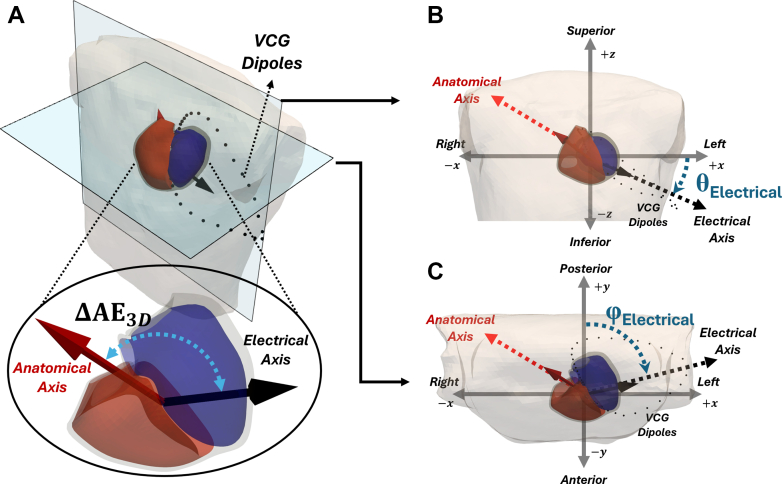
Anatomical and Electrical Axis Definitions and Angle Conventions (A) Torso schematic with coronal and transverse reference planes showing the anatomical axis (biventricular long axis) and the electrical axis (dominant QRS dipole), with example VCG dipoles. The 3D separation between anatomical and electrical axes is denoted: ΔAE_3D_; the inset indicates the region used for the exploded view. (B) Frontal-plane projection illustrating θ: tilt of the electrical axis within the frontal plane. (C) Transverse-plane projection illustrating *φ*: anterior–posterior orientation of the electrical axis in the transverse plane. VCG = vectorcardiogram.

Firstly, the anatomical axis (*v*_*A*_) was defined as the vector extending from the apex to the spatial center of the 4 valve annuli (using reconstructed cMRI meshes), and the electrical axis (*v*_*E*_) as the QRS dipole with maximum magnitude (Figure 2). The spatial separation between these axes was quantified as the 3D angle between them, ΔAE_3D_. To interrogate the contributions of each axis component, both $v_{A}$ and $v_{E}$ were expressed as spherical coordinates, denoted as *θ*_Anatomical_, *φ*_Anatomical_, *φ*_Electrical_, and *θ*_Electrical_. In anatomical terms, θ describes the tilt of the axis within the frontal plane, whereas *φ* characterizes its anterior-posterior orientation in the transverse plane. Because the anatomical axis represents cardiac orientation as a line in 3D space, all group comparisons and conclusions based on the derived angular metrics are independent of the chosen vector direction. Further details of the derivation of the 3D electrical axis from the vectorcardiogram, its corresponding planar components, and the open-access implementation used to compute these angles are available in our previous work.^11^

### Statistical analysis

Baseline characteristics are reported as mean ± SD for continuous variables, and percentages for categorical variables. These, in addition to volumetric imaging phenotypes, were compared across 3 groups: event-free population, participants with a diagnosis of LBBB before baseline (prevalent) assessment, and those who developed a diagnosis of LBBB after baseline (incident). Differences between groups were assessed using the Mann-Whitney U test and the chi-square test for continuous and categorical variables, respectively. Standard mean differences were provided in the Supplementary Material. Comparisons of baseline characteristics between groups were performed as exploratory analyses; no formal adjustment for multiple comparisons was applied.

Kaplan-Meier analyses were conducted to estimate the cumulative incidence of LBBB across population-based tertiles of QRSd and electroanatomical axis metrics. Tertile stratification was used as an exploratory approach to examine risk distribution across continuous metrics. Receiver operating characteristic analysis was performed to estimate optimal cutoffs for QRSd and *φ*_Electrical_, which aligned with the population-based tertiles. Differences between survival curves were assessed using the log-rank test.

To investigate the association between axial metrics and the incidence of LBBB, multivariable Cox proportional hazards regression models were employed. Covariates included were selected based on univariate models, clinical relevance, evidence from previous literature, and notable differences observed in group comparisons. HRs and 95% CIs were estimated to quantify the risk associated with each predictor. Harrell C-statistic was used to evaluate the discriminative performance of the model. The predictor variables were age, sex, LV ejection fraction, QRSd, hypertension, and axial metrics (*φ*_Anatomical and_ *φ*_Electrical_; based on previous analysis). Schoenfield residuals were used to assess the proportional-hazards assumption.

Based on the initial analysis, the adjunctive predictive value of *φ*_Electrical_ beyond that of QRSd was assessed. Subsequent analyses focused on *φ*_Electrical_ as it is derivable from the standard 12-lead ECG without requiring imaging. Participants were stratified into 4 groups based on whether their QRSd and *φ*_Electrical_ values were above or below predefined thresholds (tertile associated with the highest incidence of LBBB in the Kaplan-Meier analysis). Population-level projections for LBBB-ECGs performed amongst adults ≥50 were derived from primary-care ECG rates and demographic counts to estimate the potential impact of targeted repeat ECGs in adults. Detailed calculations are provided in the Supplemental Methods.

Statistical analyses were conducted in Python (3.11.7), using lifelines 0.30.0 for survival analysis and SciPy 1.13.1 for numerical computations. Statistical significance was defined as *P* value <0.05.

## Results

### Baseline characteristics

Table 1 summarizes the baseline characteristics of the reclassified study population. Compared with the event-free population, participants who developed incident LBBB were older, had slightly longer QRSd, and higher prevalence of hypertension. Notably, *φ*_Anatomical_, *φ*_Electrical_, and ΔAE_3D_ were significantly lower among those who developed LBBB compared to the event-free population. Sex distribution did not differ between groups. Decomposition of ΔAE_3D_ into the 3 anatomical planes showed that differences between the incident LBBB and event-free groups were present in the transverse (*P* < 0.001) and sagittal (*P* = 0.008) planes, but not in the frontal plane (*P* = 0.94). Further analyses focused on the transverse-plane metrics, as they remained significant in multivariable analysis.

**Table 1:** Baseline Characteristics of the Study Population (After Cardiologist-Review Reclassification), Stratified Into Participants With LBBB Diagnosed Before Assessment (Prevalent), Those Who Developed LBBB During Follow-Up (Incident), and the Event-Free Population

	Prevalent LBBB (n = 43)	Incident LBBB (n = 41)	Event-Free Population (n = 35,688)	*P* Values		
				Prevalent-Incident	Prevalent-Event-Free	Incident-Event-Free
Age (y)	69.1 ± 6.8	69.2 ± 6.0	64.0 ± 7.7	0.95	<0.001	<0.001
BMI (kg/m^2^)	26.7 ± 3.3	27.9 ± 4.3	26.1 ± 4.3	0.251	0.147	0.006
Male	48.80%	46.30%	45.20%	0.992	0.743	0.998
HTN (%)	51.20%	31.70%	11.60%	0.113	<0.001	<0.001
QRS duration (ms)	138.7 ± 15.0	90.2 ± 10.8	87.3 ± 12.7	<0.001	<0.001	0.034
LVEF (%)	49.0 ± 7.1	54.0 ± 8.7	56.0 ± 6.1	0.004	<0.001	0.300
Electroanatomical axes						
ΔAE_3D_ (°)	92.5 ± 21.4	130.5 ± 26.4	141.2 ± 24.9	<0.001	<0.001	<0.001
ΔAE_Frontal_	164.7 ± 42.9	170.9 ± 33.8	167.3 ± 30.8	0.442	0.089	0.942
ΔAE_Sagittal_	65.3 ± 26.5	109.2 ± 38.1	123.2 ± 45.2	<0.001	<0.001	0.008
ΔAE_Transverse_	85.4 ± 16.9	126.0 ± 27.5	138.2 ± 33.1	<0.001	<0.001	<0.001
$\theta_{Anatomical}$ (°)	146.0 ± 8.8	144.6 ± 9.4	143.1 ± 9.3	0.452	0.031	0.318
$\theta_{Electrical}$ (°)	−18.7 ± 43.3	−26.3 ± 31.3	−24.3 ± 31.0	0.352	0.071	0.396
*φ*_Anatomical_ (°)	65.5 ± 7.3	63.7 ± 7.7	67.0 ± 7.4	0.347	0.151	0.006
*φ*_Electrical_ (°)	33.3 ± 13.6	69.1 ± 20.6	80.3 ± 20.3	<0.001	<0.001	0.011
CMR-derived metrics^19^						
LVEDVI (mL/m^2^)	85.3 ± 15.1	75.7 ± 12.5	78.2 ± 12.9	0.007	0.009	0.270
Indexed LV Mass (g/m^2^)	47.3 ± 6.9	47.2 ± 9.4	44.9 ± 7.9	0.557	0.046	0.197
Maximum LAVI (mL/m^2^)	37.4 ± 10.8	39.2 ± 13.0	38.4 ± 10.4	0.642	0.689	0.921
Minimum LAVI (mL/m^2^)	13.5 ± 6.6	16.9 ± 8.6	15.2 ± 6.5	0.093	0.184	0.242

Values are mean ± SD for continuous variables and percentages for categorical variables. Standardized mean differences are reported in Supplemental Table 4, and support the pattern observed in the univariate comparisons.

3D = 3-dimensional; BMI = body mass index; CMR = cardiac magnetic resonance; HTN = hypertension; LAVI = left atrial volume index; LV = left ventricular; LVEDVI = left ventricular end-diastolic volume; LVEF = left ventricular ejection fraction.

There were no significant differences in left atrial parameters between groups. Indexed LV end-diastolic volume was significantly larger in the prevalent LBBB group compared to both the incident LBBB group (+13%; *P* = 0.007) and the event-free population (+9%, *P* = 0.009), whereas the incident LBBB group showed no significant difference from event-free participants (*P* = 0.27). Indexed LV mass was marginally higher in the prevalent LBBB group (+5.3%; *P* = 0.046) and the incident LBBB group (*P* = 0.197) compared to the event-free population. The contrasting presence and lack of structural changes in the prevalent and incident groups indicate that, in this cohort at the time point assessed, there was no structural remodeling preceding LBBB onset.

### Associations with incident LBBB

Electrical axis metrics demonstrated progressive posterior deviation preceding LBBB development, with incident cases showing axis values overlapping with prevalent LBBB before diagnostic QRS prolongation (Supplemental Figure 1). Frontal plane axes were not associated with LBBB development, and they were not considered for further analysis. During follow-up, only 4 event-free participants (0.01%) received a new LAFB diagnosis, none of whom developed LBBB.

**Figure 3:**
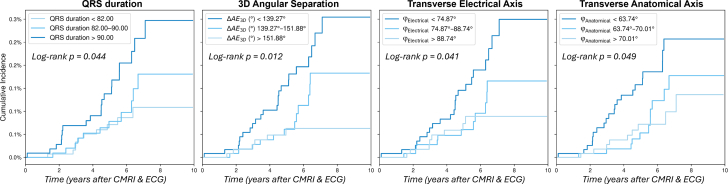
Kaplan-Meier Plots of the Primary Endpoint, Incident Left Bundle Branch Block, Stratified by Population-Based Tertiles of QRS Duration, ΔAE_3D_, and Transverse Electroanatomical Axes Darker color represents tertile threshold per metric. 3D = 3-dimensional; other abbreviations as in Figure 1.

The Kaplan-Meier plots demonstrate that QRSd and the transverse axes (*φ*_Electrical_ and *φ*_Anatomical_) were associated with the cumulative incidence of LBBB (Figure 3). Individuals in the highest tertile of QRSd and the lowest tertile of *φ*_Electrical_ exhibited a markedly increased incidence of LBBB (log-rank *P* < 0.05), with a slightly weaker separation in *φ*_Anatomical_. This indicates a more posterior electrical axis link with higher cumulative incidence.

**Figure 4:**
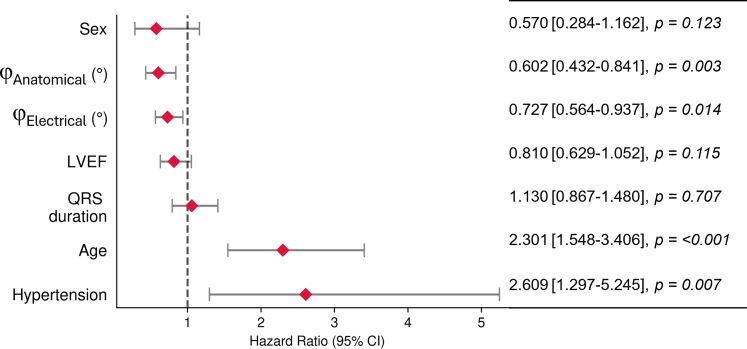
Forest Plot of the Multivariable Cox Proportional-Hazards Model Using Left Ventricular Ejection Fraction, Hypertension, Age, Sex, *φ*_Electrical_, and QRS Duration for Incident Left Bundle Branch Block The plot shows HRs per SD change except for hypertension and sex. C-statistic = 0.82. Internal validation using repeated stratified 5-fold cross-validation (10 repeats) yielded a cross-validated C-statistic of 0.790 (95% CI: 0.674-0.910). LVEF = left ventricular ejection fraction.

Multivariable Cox regression (Figure 4) identified *φ*_Anatomical_, *φ*_Electrical_, age, and hypertension as independent predictors of LBBB development. After clinical and demographic covariate adjustment, *φ*_Electrical_ (HR per SD decrease: 0.73 [0.56-0.94]; *P* = 0.014) showed significant association with LBBB development, but QRSd did not. Likelihood ratio testing confirmed the incremental predictive value of φ_Electrical_ and φ_Anatomical_ beyond QRSd and clinical covariates (chi-square = 13.23; df = 2; *P* = 0.0013). Schoenfeld residuals confirmed that the proportional hazards assumption was satisfied for all covariates (all *P* > 0.05).

### Combined stratification of QRS duration and transverse electrical axis

**Figure 5:**
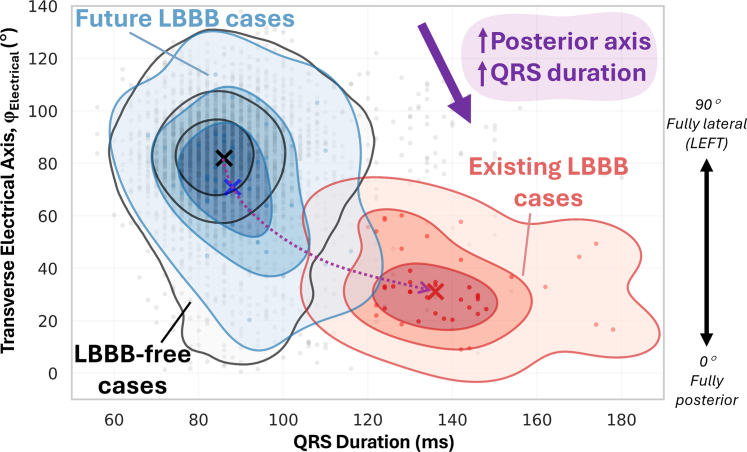
Distribution of QRS Duration and Transverse Electrical Axis by Left Bundle Branch Block Status at Baseline Kernel density estimates show more posterior electrical axes and longer QRS durations in the incident LBBB group (blue). Dashed arrow demonstrates hypothesized transition to LBBB. Contour lines denote probability-density levels, and crosses mark the median of each group. Abbreviation as in Figure 1.

**Figure 6:**
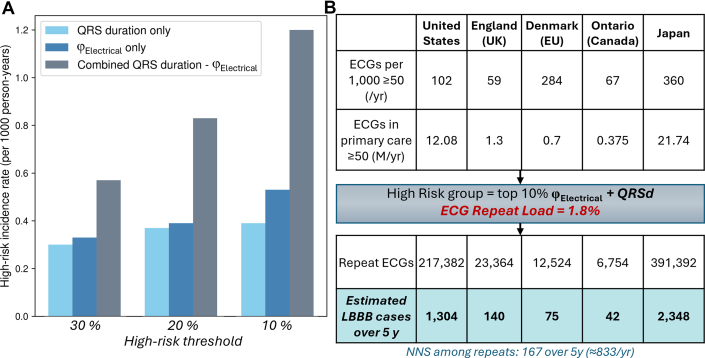
Population-Level Screening Utility of Combined Risk Stratification (A) Incidence rates within UK Biobank in high-risk groups defined by the top 30%, 20%, and 10% of each metric (direction harmonized so higher values indicate greater risk). ROC-derived optimal cutoffs for QRS duration and *φ*_Electrical_ are provided in the Supplementary Materials and were aligned with the population-based thresholds used for stratification. (B) Regional estimates for implementation in primary care (≥50-year-old adults) with the top 10% combined thresholds. Repeat ECGs are initial recalls per year (based on the middle-aged, predominantly white UK Biobank cohort). NNS = number needed to screen; other abbreviations as in Figure 1.

The baseline distribution of QRSd and *φ*_Electrical_ values illustrates directional shifts in both parameters among participants who subsequently developed LBBB (Figure 5). Joint stratification by QRSd and *φ*_Electrical_ tertiles demonstrates a consistent multiplicative effect (Central Illustration, Figure 6A). Individuals in the arbitrary highest risk group (highest QRSd and lowest *φ*_Electrical_ tertile) showed a substantially higher LBBB incidence rate of 0.62 vs <0.2 per 1,000 person-years compared to the other groups, with an HR of 4.09 (1.84-8.99, *P* < 0.001) after covariate adjustment, indicating that QRSd and *φ*_Electrical_ represent complementary markers of an increased risk of future LBBB. Figure 6 demonstrates population-level incidence rates and estimated projections for potential screening utility.

Exploratory sex-stratified analysis showed ∼40% higher incidence of LBBB among high-risk females compared to males (0.76 vs 0.53 per 1,000 person-years), consistent with evidence of females having more posterior transverse electrical axis^11^ but a lower baseline QRSd.^20^

## Discussion

This analysis of 35,749 UK Biobank participants demonstrates that a posterior shift in the transverse electrical axis, *φ*_Electrical_, derived from the standard 12-lead ECG, identifies subclinical conduction changes that precede the development of LBBB pattern on ECG. When combined with QRSd, it identifies individuals at an increased risk of future development of LBBB, providing a unique opportunity for early identification of this group that outperforms conventional 1-dimensional ECG metrics.

### Mechanistic insights of posterior electrical axis shift

**Figure 7:**
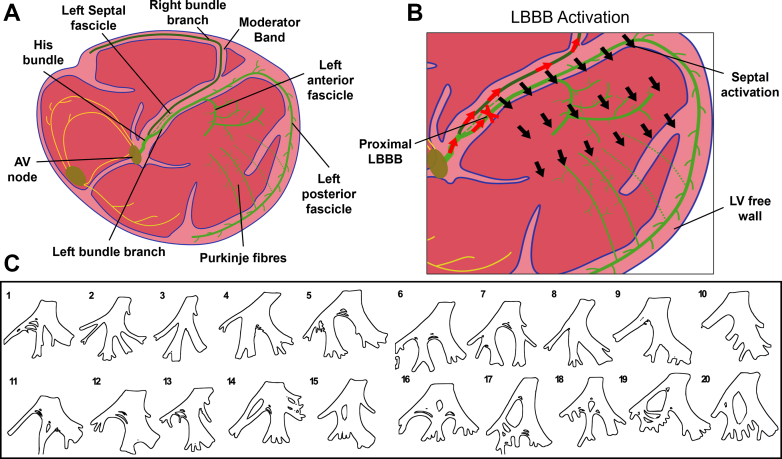
Cardiac Conduction System and Anatomical Representation of Electrical Axis (A) Schematic illustration of the cardiac conduction system. (B) Electrical impulse propagation (red arrows) from the His bundle with a proximal LBBB showing RBB activation of the septum followed by myocardial activation towards the LV free wall (black arrows). (C) Left-sided conduction system diagrammatic sketches from 20 human heart transverse sections.

Reproduced from Histopathological examination of concept of left hemiblock, Demoulin JC, Kulbertus HE, Volume 34, pages 807 to 814, copyright 1972, with permission from BMJ Publishing Group Ltd.^22^ AV = atrioventricular.

Established LBBB is sometimes the result of focal disruption of conduction at the proximal LBB;^1^^,^^21^ however, intracardiac mapping has demonstrated that LBBB-pattern morphologies encompass a spectrum of conduction abnormalities, including conduction failure at the proximal LBB, diffuse distal LV conduction system delay, and even postconduction system intramyocardial conduction delay.^3^ In the case of proximal conduction block in the LBB, the RBB activates the septum, resulting in an activation wavefront toward the LV free wall that introduces septal-free wall dyssynchrony.^7^ This delayed LV free-wall activation alters the dominant electrical dipole and explains why established LBBB typically drives a posterior shift of the transverse electrical axis (Figure 7B, Central Illustration).

**Central Illustration:**
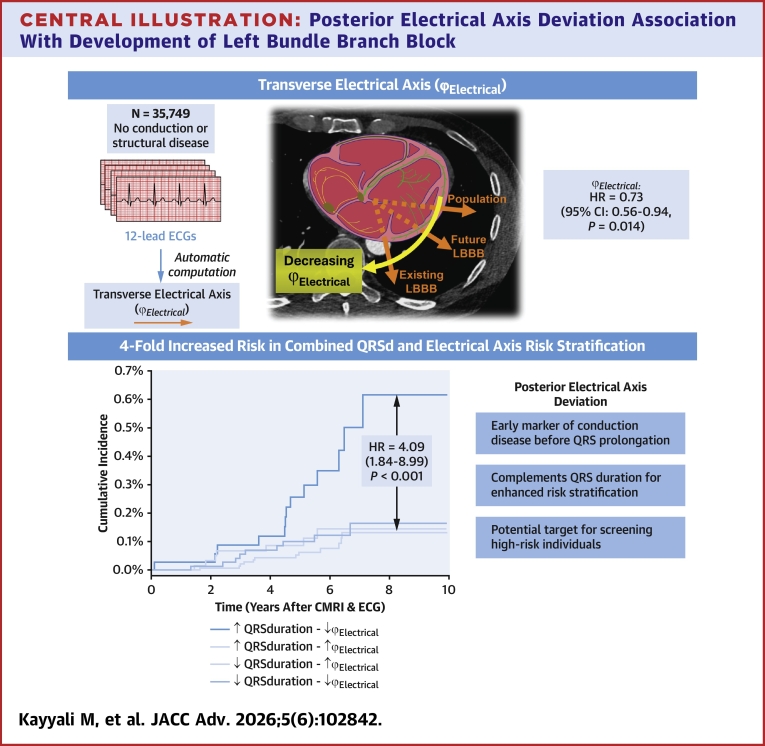
Posterior Electrical Axis Deviation Association With Development of Left Bundle Branch Block Transverse electrical axis (*φ*_Electrical_) is automatically extracted from 12-lead ECGs. Combined with QRS duration, *φ*_Electrical_ provides multiplicative risk enhancement (high-risk individuals with 4-fold increased LBBB incidence), presenting a potential early marker of progressive conduction deterioration before diagnostic QRS prolongation. ECG = electrocardiogram; LBBB = left bundle branch block; QRSd = QRS duration.

### Potential pathophysiological mechanisms of progressive conduction-system change

Our findings demonstrate that in a middle-aged population without pre-existing cardiac disease, early, measurable changes, predominantly in the transverse cardiac electrical axis and prior to an increase in QRSd above widely accepted normal ranges, predict the future development of LBBB. In this population, the LBBB pattern on ECG may be the result incremental conduction changes in conduction system behavior, suggesting a progressive alteration in left-sided conduction rather than an abrupt, focal event as the dominant pathway in this cohort.

#### Anatomical substrate and conduction system variability

The LBB emerges from the inferior border of the membranous septum and divides into distinct fascicular entities: a thin anterior fascicle, a wider left posterior fascicle (LPF) and sometimes a septal fascicle (Figure 7A).^12^^,^^22^ However, considerable variation exists in the normal human heart (Figure 7C) with diversity in the arborization between fascicles and variability in the width, length, and degree of branching.^23^^,^^24^ This anatomical heterogeneity creates differential vulnerability patterns, especially as the LPF’s broad distal branching towards the posterior LV free wall provides a larger target for diffuse compromise (eg, fibrosis), which is consistent with a posterior deviation of the electrical axis.

LAFB is a common incidental finding on ECG that is usually considered to be benign and existing data does not indicate that LAFB represents a precursor to LBBB.^23^^,^^25^ In this cohort, no participant who developed incident LBBB had a fascicular block diagnosis at baseline or during follow-up. The axis deviation observed in the incident LBBB group was confined to the transverse plane, with no significant frontal-plane axis difference. This dissociation is inconsistent with typical fascicular block patterns (LAFB: leftward frontal-axis deviation, left posterior fascicular block: rightward/superior deviation). Instead, the findings indicate a conduction abnormality better reflected in the transverse plane, possibly preceding ECG-detectable fascicular block. We hypothesize that the posterior electrical axis deviation may reflect a diffuse and progressive process affecting the left-sided conduction system, indicating intermediate time points between healthy or “normal” conduction system behavior and the presence of frank LBBB. This hypothetical progression would represent a contrast with abrupt presentations such as LBBB after transcatheter aortic valve intervention, where focal conduction block is precipitated by procedural trauma.^24^

#### Potential mechanisms

With the hypothesis that diffuse conduction system alterations may be the predominant mechanism underlying LBBB, rather than focal fascicular block, in this cohort, there are several plausible possibilities for the mechanisms underlying this process. With advancing age progressive fibrosis and calcification occurs affecting structures adjacent to the conduction system,^1^ and these may affect multiple fascicular components simultaneously. This would be supported by the observed association between hypertension and incident LBBB, which could result from hypertension-mediated conduction-system fibrosis, in which adverse hemodynamic load resulting from hypertension activates profibrotic pathways (eg, angiotensin II, transforming growth factor-β), promoting collagen deposition.^26^

However, before diagnostic LBBB manifests, regional changes in conduction/activation can already produce posterior shifts of the transverse electrical axis (Central Illustration), providing a mechanistic bridge from subtle axis deviation to overt dyssynchrony. The transition toward loss of electrical synchrony could be driven by several, not mutually exclusive, processes.

i) Distal compromise (fibrosis/metabolic stress): Delays, likely concentrated in LPF-supplied regions, may shift the axis posteriorly before QRSd widens. If a fraction of distal LPF fibers becomes nonconducting (eg, ∼30%/70%), activation proceeds through fewer parallel pathways with a lower safety factor and longer local conduction times.^27^, ^28^, ^29^
ii) Purkinje-myocardial junction (PMJ) remodeling: Reduced connexin-43 or slowed junctional conduction at distal Purkinje-myocardial junctions may preferentially delay activation of the posterior free wall, incrementally increasing dyssynchrony while QRSd remains within normal limits.^27^^,^^29^
iii) Patchy fascicular/intrahisian microscarring,^29^^,^^30^ causing lower functional *reserve:* Given the longer path length and greater distal branching of the LPF (Figure 7C), loss of a subset of fibers possibly leaves fewer functional pathways posteriorly, reduces transverse current flow, and produces an early posterior axis shift. With progression, global QRSd would then increase.

Consistent with an early conduction-system process, baseline chamber sizes and LV mass were preserved in the incident LBBB group (Table 1), despite LBBB typically being associated with progressive LV remodeling and volumetric change in other contexts.^31^^,^^32^

### Clinical implications

The complex role of LBBB in cardiac pathology manifests through 3 interconnected mechanisms: as an instigator of electromechanical dysfunction, a secondary consequence of remodeling, and an amplifier of pre-existing compromise. As a *cause*, LBBB induces dyssynchronous ventricular activation that promotes adverse remodeling, HF progression, and LBBB-induced CM.^21^^,^^29^ Conversely, LBBB frequently emerges as a *consequence* of subclinical fibrosis or ischemic injury within the conduction system, serving as an electrophysiological biomarker of advancing myocardial disease.^1^^,^^32^ LBBB acts as an *exacerbator* by creating feedback loops that accelerate electrical-mechanical dissociation, including worsening mitral regurgitation and impairing diastolic function.^31^ This triad of roles underscores the value of metrics that quantify early, evolving abnormalities.

The transverse electrical axis is a measurable, automatable, and scalable ECG metric for identifying future LBBB risk from routine recordings, requiring only digital ECGs and standard programming tools. The anatomical axis was included to account for the contribution of cardiac orientation to the electrical axis deviation. Both *φ*_Electrical_ and *φ*_Anatomical_ were independently significant in multivariable analysis. The 3D angular separation (ΔAE_3D_) was also significant (Supplemental Table 3) but did not improve performance. This suggests that cardiac orientation contributes to the observed axis pattern, but to a lesser and less consistent extent than the electrical shift itself (Table 1, Supplemental Figure 1). The basis of this anatomical association remains uncertain. Subsequent analyses therefore focused on *φ*_Electrical_.

In our cohort, incidence increased stepwise with stricter QRSd-axis thresholds (Figure 6A), with joint stratification showing a multiplicative effect. Operationally, this supports targeted repeat ECGs in primary care settings for the highest-risk groups (Figure 6B), which would be especially relevant in certain groups such as hypertensive populations.^33^ Before the development of frank LBBB, the transverse electrical axis appears a more sensitive marker of incipient conduction disease than QRSd. This may have implications for patient selection in non-standard CRT indications (eg pre-LBBB in borderline QRS with early conduction abnormalities), complementing reports that horizontal electrical axis better predicts CRT response in patients with existing LBBB.^10^

#### Potential for early identification of high-risk populations

To establish whether these metrics have the potential to identify a higher risk cohort and therefore operate as screening tools, progressively stricter thresholds (top 30%, 20%, 10%) were applied (Figure 6A). Incidence rose stepwise (0.58, 0.83, and 1.2 per 1,000 person-years), and the *combined* metric consistently outperformed QRSd or *φ*_Electrical_ alone.

Risk thresholds were applied to international health care systems with varying ECG utilization patterns to illustrate potential for population-level screening implications (Figure 6B). The top 10% combined risk threshold would prompt recall of 1.8% of patients following baseline ECG with a 5-year risk of incident LBBB among recalled individuals of 0.60%. This yields a *number needed to screen* among repeats of 167. If implemented in this manner this approach would identify about 108 patients with LBBB per million baseline ECGs, and prompt ∼18,000 repeat ECGs per million baseline ECGs. Moving from the 10% to the 20% threshold increases repeat testing by roughly 3.2-fold and increases detected LBBB by about 2.2-fold. Appropriate thresholds would need to reflect detailed consideration of healthcare benefits of early identification. These *estimated* counts are specifically based on the UK Biobank cohort (middle-aged, predominantly white, healthy bias) and numbers may differ in more diverse or higher-risk populations. It should be noted that these estimates are illustrative and intended to contextualize potential operational impact and are not intended as screening recommendations.

### Study limitations

This study’s findings should be interpreted in light of several limitations. The predominantly White ethnicity (96%) limits generalizability to diverse populations, particularly given potential genetic contributions to conduction system disease. However, ICD-10-based outcome ascertainment was the most feasible approach in an exploratory large population-based cohort study. Because numerical estimates arise from a middle-aged, predominantly White UK Biobank cohort, the absolute yields and workload projections are population-specific and may be higher, and differently distributed, in more diverse or higher-risk settings. The small number of incident LBBB events limited statistical power and precluded extensive subgroup analyses. The low event rate likely reflects the use of a general population free of baseline cardiovascular disease and the healthy volunteer bias of the UK Biobank.^34^ Nevertheless, the main findings were consistent across multiple analytical approaches, and the multivariable model was restricted to clinically relevant covariates to reduce the risk of overfitting and instability. Reliance on hospital ICD-10 coding may have led to underestimation of incident LBBB, particularly for cases diagnosed outside hospital settings or not formally documented. There was a lack of interobserver variability assessment during the ECG review process, but that is minimized by strictly following the ESC criteria statements. The exclusion of participants with baseline cardiovascular disease, whereas methodologically sound for studying incident LBBB, limits applicability to higher-risk clinical populations where this tool may have greatest utility. Reliance on retrospective ICD-10 coding for LBBB diagnosis depends on the diagnostic accuracy of the clinicians making these judgments which are not subject to quality control. Subclinical or undocumented fascicular conduction abnormalities cannot be excluded as possible precursors to LBBB because follow-up diagnoses were based on routine clinical records. Although axis metrics provides a global measure of electrical propagation, it does not localize the precise site of conduction delay, such as distinguishing proximal from distal left bundle lesions, which limits mechanistic specificity. There is also the potential for residual confounding from unmeasured comorbidities or medication use.

### Future directions

Future research should aim to validate the predictive value of axis-based metrics in diverse cohorts with established CM, postmyocardial infarction, or other structural heart diseases. Longitudinal studies tracking serial changes in axis orientation could clarify their relationship with the onset and progression of LV dysfunction. Prospective, sex-stratified studies are needed to determine whether thresholds should be tailored for female patients, given emerging evidence of sex-specific conduction pathology (sex-stratified analyses presented in the current study are to be considered hypothesis-generating*)*. Finally, integrating these metrics with artificial intelligence-driven ECG analysis represents a natural evolution toward precision, state-of-the-art electrocardiology.

## Conclusions

In middle aged adults without overt cardiovascular disease, the transverse electrical axis calculated from 12-lead ECG represents a metric which may identify individuals with early conduction system remodeling who are at risk of future development of LBBB. This has the potential to operate as a simple and low-cost screening tool that could be easily deployed. Prospective validation is warranted to confirm its utility in guiding surveillance and therapeutic strategies.

Perspectives **COMPETENCY IN MEDICAL KNOWLEDGE:** The transverse electrical axis, derived from the standard 12-lead ECG, may identify subclinical conduction system disease before QRSd exceeds normal limits.

**COMPETENCY IN SYSTEMS-BASED PRACTICE:** *φ*_Electrical_ calculation is fully automatable in digital ECG systems, requiring no manual measurements or additional testing.

**TRANSLATIONAL OUTLOOK:** External validation in ethnically diverse and higher-risk populations, with prospective studies using adjudicated LBBB outcomes, is needed to confirm generalizability, refine risk thresholds, and strengthen diagnostic accuracy. Clinical implementation will require integration into commercial ECG systems, standardized reference ranges, and evaluation of whether identifying high-risk individuals can improve outcomes.

## Funding support and author disclosures

Kayyali was supported by King’s College London Centre for Doctoral Training in Digital Twins for Healthcare. Dr Mincholé acknowledges support from the 10.13039/501100004837MCIN/AEI/10.13039/501100011033 through grants PID2021 to 128972OA-I00, TED2021 to 130459B-100, CNS2022 to 135899, and the RYC2019 to 027420-I fellowship. Dr Qian was supported by the 10.13039/501100023312Wellcome/EPSRC Centre for Medical Engineering (WT203148/Z/16/Z). Dr Bishop acknowledges support from the 10.13039/501100000274British Heart Foundation (BHF) through Project Grants PG/22/10871, PG/22/11159, and PG/18/74/34077. All other authors have reported that they have no relationships relevant to the contents of this paper to disclose.

## Appendix

Supplemental Material

Supplemental Permissions for Figure
