# Supplementary material for: Posterior Electrical Axis Deviation Is Associated With Future Development of Left Bundle Branch Block: A UK Biobank Study
Source: JACC Adv. 2026 Jun 17;5(6):102842. doi: 10.1016/j.jacadv.2026.102842 (PMC13308251; doi:10.1016/j.jacadv.2026.102842)
Supplement: Supplemental Material [file mmc1.pdf]

Supplemental Material

Supplemental Methods

1. Data Acquisition & Processing

Each participant's 10-second recording was sampled at 500 Hz with a 1 µV resolution and underwent low-pass (100 Hz), high-pass (0.1 Hz) and 50 Hz power-line filters. No additional preprocessing was applied. A median beat was extracted for subsequent analysis, and ECGs were excluded if their QRS amplitude (maximum deflection from isoelectric line) fell outside ±3 standard deviations of the population mean for each lead.

The median ECG was converted into a vectorcardiogram (VCG) using the Kors transformation, producing three orthogonal leads: V_x, V_y, and V_z. This approach captures the heart vector's transient behaviour, showing how the cardiac dipole changes in both magnitude and orientation throughout the cardiac cycle.

2. Data Classification

Supplementary Table 1. Classification of UK Biobank Participants by ICD-10 hospital coding followed by reclassification after manual review of a cardiac electrophysiologist

Original classification	Reclassification	N	Final group
**Prevalent LBBB (N=48)**	Confirmed LBBB	23	Prevalent LBBB
	Normal	12	Excluded
	Partial LBBB	6	Excluded
	RBBB	3	Excluded
	LAFB	1	Excluded
	Other	3	Excluded
**Incident LBBB (N=65)**	LBBB at baseline	20	Reclassified as prevalent
	Normal baseline ECG	41	Incident LBBB
	RBBB	2	Excluded
	Partial LBBB	2	Excluded
**Final groups**	Prevalent LBBB	43	(23 + 20)
	Incident LBBB	41	

3. Population-level estimates

Purpose: We projected the potential impact of targeted repeat ECGs in adults ≥50 by region. For each region we estimated the annual repeat-test workload and the 5-year yield of incident LBBB under the top-10% combined threshold and the top-20% threshold.

Metrics (common across regions)

1. Baseline ECG throughput in primary care (≥50).
   - Annual ECG rate per 1,000 ≥50 and the ≥50 population for each region
   - converted rates to counts by multiplying the rate by the population and dividing by 1,000.
2. Repeat-test fraction (recalls).

    - Top 10%: 1.8% of all baseline ECGs (observed in UK Biobank).
    - Top 20%: 5.7% of baseline ECGs.
3. Event risk among recalled individuals (LBBB).
    - Top 10%: 0.60% 5-year cumulative incidence of incident LBBB among those recalled.
    - Top 20%: 0.42% 5-year cumulative incidence.
4. Number needed to screen (NNS) among repeats (for context).
    - Top 10%: 167 repeat ECGs per LBBB over 5 years.
    - Top 20%: 238 repeat ECGs per LBBB over 5 years.
    - These values are simply the reciprocal of the 5-year risks above and are invariant across regions.

Calculation steps (applied identically to each region)

- Step 1: Baseline ECGs/year (≥50).
  Annual primary-care ECGs = *(rate per 1,000 ≥50) × (population ≥50 / 1,000)*.
- Step 2: Repeat ECGs (initial recalls) per year.
  Multiply baseline ECGs by 1.8% (top-10%). For the 20% scenario, used 5.7%.
- Step 3: Estimated LBBB cases over 5 years (among repeats).
  Multiply repeat ECGs (from Step 2) by the 5-year risk among recalled individuals (top-10%: 0.60%; top-20%: 0.42%).
  Interpretation: these are cases expected over 5 years arising from a single year's recalls.
- Reporting conventions.
  Baseline ECGs are shown in millions; repeat ECGs and LBBB counts are whole numbers. Ranges reflect scenario changes only (top-10% vs top-20%).

Region-specific inputs and notes (as cited in Methods)

United States [based on national claims, primary care]

- Rate: 102 per 1,000 ≥50 (primary care). Population ≥50: 118.4 million.
- Baseline ECGs/year = 102 × 118.4M ÷ 1,000 = 12,076,800.
- Repeat ECGs/year (top-10%) = 1.8% × 12,076,800 = 217,382.
- Estimated LBBB over 5 y (top-10%) = 0.60% × 217,382 = 1,304.
- Notes on rate derivation: Medicare Part B CPT 93000 volumes (≥65) calibrated to ≥50 and restricted to primary care using an assumed ~45% primary-care share; ≥50 population from U.S. Census.

England (UK) [derived from GP diagnostic activity]

- Rate: 70 per 1,000 ≥50. Population ≥50: 22.0 million.
- Baseline ECGs/year = 70 × 22.0M ÷ 1,000 = 1,540,000.
- Repeat ECGs/year (top-10%) = 1.8% × 1,540,000 = 27,720.
- Estimated LBBB over 5 y (top-10%) = 0.60% × 27,720 = 166.
- How the rate was obtained: O'Sullivan et al. (2018) report all-age GP ECG activity ≈34.7 per 1,000. We multiplied by 2 to reflect higher consultation intensity in ≥50, giving 70 per 1,000. Population from ONS mid-year estimates.

Denmark (EU) [regional primary-care laboratory counts]

- Rate: 284 per 1,000 ≥50. Population ≥50: 2.45 million.
- Baseline ECGs/year = 284 × 2.45M ÷ 1,000 = 695,800.
- Repeat ECGs/year (top-10%) = 1.8% × 695,800 = 12,524.

- Estimated LBBB over 5 y (top-10%) = 0.60% × 12,524 = 75.
- How the rate was obtained: CopLab all-age rate ≈142 per 1,000; we approximated ≥50 as ~2× all-age → 284 per 1,000. This is a regional estimate, not national. Population from Statistics Denmark.

Ontario (Canada) [AHE-only, upper-bound uptake; ignores non-AHE ECGs]

- Rate (AHE-only): ≈67 per 1,000 ≥50. Population ≥50: 5.6 million.
- Baseline ECGs/year = 67 × 5.6M ÷ 1,000 = 375,200.
- Repeat ECGs/year (top-10%) = 1.8% × 375,200 = 6,754.
- Estimated LBBB over 5 y (top-10%) = 0.60% × 6,754 = 41.
- How the rate was obtained: Bhatia et al. (2017) found 21.5% of annual health examinations (AHEs) include an ECG within 30 days. Saunders et al. (2019) reported an upper-bound AHE uptake ~31% in Ontario. We multiplied 0.31 × 0.215 × 1,000 = 67 per 1,000, and deliberately ignored ECGs at non-AHE visits, so this is a lower-bound population estimate by design. Population from Statistics Canada.

Japan [working-age health-check proxy applied pragmatically to ≥50]

- Rate: ≈360 per 1,000 ≥50. Population ≥50: 60.4 million.
- Baseline ECGs/year = 360 × 60.4M ÷ 1,000 = 21,744,000.
- Repeat ECGs/year (top-10%) = 1.8% × 21,744,000 = 391,392.
- Estimated LBBB over 5 y (top-10%) = 0.60% × 391,392 = 2,348.
- How the rate was obtained: Yagi et al. (2024) show resting ECG is routinely included in the annual worker health check. Their flow diagram reports 5,442,529 ECGs among 15,005,920 enrolees in 2015, which is ≈363 per 1,000 in insured ages 35–65. Because a comprehensive ≥50 primary-care ECG rate is not published, we apply this observed working-age rate to the ≥50 denominator and round to 360 per 1,000. This anchors the estimate to in-paper counts while acknowledging participation above retirement age differs. Population from the Statistics Bureau of Japan.

Top-20% threshold

To show the trade-off between recall volume and yield, we repeated Step 2 and Step 3 using a 5.7% repeat fraction and 0.42% 5-year LBBB risk. This increases repeat testing by about 3.2× compared with the top-10% rule and increases expected LBBB detections by about 2.2×. NNS among repeats under this scenario is 238 over 5 years.

Supplementary Table 2. Complete stepwise numbers used for population-level estimates for targeted screening.

Region	ECG rate per 1,000 ≥50 (pop/yr)	Population ≥50 (M)	ECGs/year in ≥50 primary care	Repeat ECGs (20% High Risk group)	LBBB estimated 20%	Repeat ECGs (10% High Risk group)	10% LBBB estimated	Key Sources
United States	102	118.4	12,076,800	688,378	2,891	217,382	1,304	Centers for Medicare & Medicaid Services. Medicare Fee-for-Service Part B Utilization, CY 2022, CPT 93000; U.S. Census Bureau. National Population by Characteristics, 2023.
England (UK)	59	22	1,298,000	73,986	311	23,364	140	O'Sullivan JW et al. BMJ 2018; Office for National Statistics. Population estimates, mid-2023.

Denmark (EU)	284	2.45	695,800	39,661	167	12,524	75	Kriegbaum M et al. Clin Epidemiol 2024 (CopLab); Statistics Denmark.
Ontario (Canada)	67	5.6	375,200	21,386	90	6,754	41	Saunders NR et al. BMC Primary Care 2019 (AHE uptake); Bhatia RS et al. JAMA Intern Med 2017 (ECG after AHE); Statistics Canada Table 17-10-0005-01.
Japan	360	60.4	21,744,000	1,239,408	5,206	391,392	2,348	Yagi R et al. JAMA Intern Med 2024; Statistics Bureau of Japan.

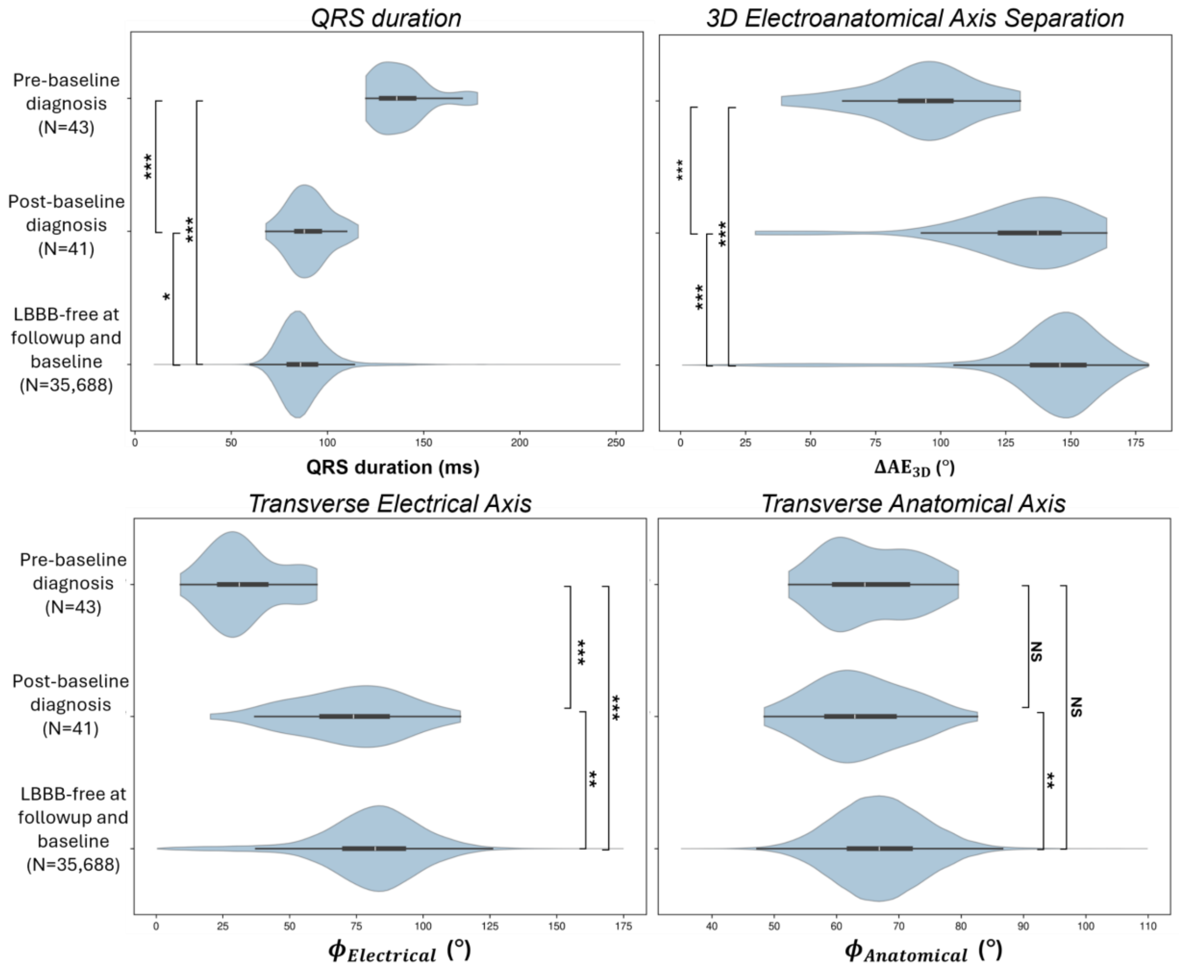

Figure 1. Distributions of QRSd and electro-anatomical axis metrics in prevalent LBBB (pre-baseline diagnosis), incident LBBB (post-baseline diagnosis) and LBBB-free cohorts. $\phi_{Anatomical}$ and $\phi_{Electrical}$ represent anterior-posterior orientations. *P-value*: *** < 0.001, ** < 0.01, * < 0.05, NS = not significant.

Supplementary Table 3. Multivariable Cox proportional hazards models for axis metrics and incident LBBB

Model	Variable	HR	95% CI	P-value
**Main model**	φ_Electrical	0.73	0.56–0.94	0.014
	φ_Anatomical	0.60	0.43–0.84	0.003
**φ_Electrical only**	φ_Electrical	0.75	0.57–0.95	0.026
ΔAE_3D	ΔAE_3D	0.79	0.63–0.99	0.037
ΔAE_Transverse	ΔAE_Transverse	0.85	0.71–1.01	0.059
ΔAE_Sagittal	ΔAE_Sagittal	0.85	0.66–1.09	0.203
ΔAE_Frontal	ΔAE_Frontal	1.24	0.89–1.72	0.206

All models adjusted for QRSd, sex, age, hypertension, and LVEF. Hazard ratios for continuous variables are per SD change.

Supplementary Table 4. Baseline characteristics with standardised mean differences between incident LBBB and event-free populations.

	Prevalent LBBB *N = 43*	Incident LBBB *N = 41*	Event-free Population *N = 35,688*	P-values			Standardised mean difference
				Prevalent-Incident	Prevalent-Event-free	Incident-Event-free	
**Age (years)**	**69.1±6.8**	**69.2±6.0**	**64.0±7.7**	**0.95**	**<0.001**	**<0.001**	**0.752**
**BMI (kg/m²)**	**26.7±3.3**	**27.9±4.3**	**26.1±4.3**	**0.251**	**0.147**	**0.006**	**0.408**
Sex (Male)	48.80%	46.30%	45.20%	0.992	0.743	0.998	0.023
**HTN (%)**	**51.20%**	**31.70%**	**11.60%**	**0.113**	**<0.001**	**<0.001**	**0.504**
**QRS duration (ms)**	**138.7±15.0**	**90.2±10.8**	**87.3±12.7**	**<0.001**	**<0.001**	**0.034**	**0.247**
LVEF (%)	49.0±7.1	54.0±8.7	56.0±6.1	0.004	<0.001	0.300	-0.260
Electroanatomical axes							
**ΔAE_{3D} (°)**	**92.5 ± 21.4**	**130.5±26.4**	**141.2±24.9**	**<0.001**	**<0.001**	**<0.001**	**-0.418**
$\Delta AE_{Frontal}$	164.7±42.9	170.9±33.8	167.3±30.8	0.442	0.089	0.942	0.11
**$\Delta AE_{Sagittal}$**	**65.3±26.5**	**109.2±38.1**	**123.2±45.2**	**<0.001**	**<0.001**	**0.008**	**-0.335**
**$\Delta AE_{Transverse}$**	**85.4±16.9**	**126.0±27.5**	**138.2±33.1**	**<0.001**	**<0.001**	**<0.001**	**-0.402**
$\theta_{Anatomical}$ (°)	146.0 ± 8.8	144.6 ± 9.4	143.1±9.3	0.452	0.031	0.318	0.166
$\theta_{Electrical}$ (°)	-18.7 ± 43.3	-26.3 ± 31.3	-24.3±31.0	0.352	0.071	0.396	-0.065
**$\varphi_{Anatomical}$ (°)**	**65.5 ± 7.3**	**63.7±7.7**	**67.0±7.4**	**0.347**	**0.151**	**0.006**	**-0.434**
**$\varphi_{Electrical}$ (°)**	**33.3 ± 13.6**	**69.1±20.6**	**80.3±20.3**	**<0.001**	**<0.001**	**0.011**	**-0.378**
CMR-derived metrics [19]							
LVEDVI (mL/m²)	85.3±15.1	75.7± 12.5	78.2±12.9	**0.007**	**0.009**	0.270	-0.278
Indexed LV Mass (g/m²)	47.3±6.9	47.2±9.4	44.9±7.9	0.557	**0.046**	0.197	0.259
Maximum LAVI (mL/m²)	37.4±10.8	39.2±13.0	38.4±10.4	0.642	0.689	0.921	0.067
Minimum LAVI (mL/m²)	13.5±6.6	16.9±8.6	15.2±6.5	0.093	0.184	0.242	0.226

References [1–12]

1. U.S. Census Bureau: National Population by Characteristics: 2023 (Single Year of Age and Sex) [Internet]. 2024,. Available from: https://www.census.gov/data/tables/time-series/demo/popest/2020s-national-detail.html

2. Centers for Medicare & Medicaid Services: Medicare Fee-for-Service Part B Utilization: Top 200 Level I CPT Codes Ranked by Charges, CY 2022 [Internet]. 2023,. Available from: https://www.cms.gov/data-research/statistics-trends-and-reports/medicare-fee-for-service-parts-a-b/medicare-utilization-part-b

3. O'Sullivan JW, Stevens S, Hobbs FDR, et al.: Temporal trends in use of tests in UK primary care, 2000-15: retrospective analysis of 250 million tests. BMJ [Internet] British Medical Journal Publishing Group, 2018 [cited 2025 Aug 13]; 363:4666. Available from: https://www.bmj.com/content/363/bmj.k4666

4. Office for National Statistics: Population estimates for England and Wales: mid-2023 [Internet]. 2024,. Available from: https://www.ons.gov.uk/peoplepopulationandcommunity/populationandmigration/populationestimates/bulletins/populationestimatesforenglandandwales/mid2023

5. Kriegbaum M, Lind BS, Grand MK, Andersen CL: The Copenhagen Primary Care Laboratory (CopLab) Database. Clin Epidemiol [Internet] Dove Medical Press Ltd, 2024 [cited 2025 Aug 13]; 16:155. Available from: https://pmc.ncbi.nlm.nih.gov/articles/PMC10913898/

6. Statistics Denmark: Population figures (by age and sex) [Internet]. 2024,. Available from: https://www.dst.dk/en/Statistik/emner/borgere/befolkning/befolkningstal

7. Bhatia RS, Bouck Z, Ivers NM, et al.: Electrocardiograms in Low-Risk Patients Undergoing an Annual Health Examination. JAMA Intern Med [Internet] American Medical Association, 2017 [cited 2025 Aug 12]; 177:1326–1333. Available from: https://jamanetwork.com/journals/jamainternalmedicine/fullarticle/2643348

8. Statistics Canada: Table 17-10-0005-01: Population estimates on July 1, by age and sex [Internet]. 2024,. Available from: https://www150.statcan.gc.ca/t1/wds/cansim/wds/cansimtabletableau?pid=1710000501

9. Ministry of Health L, (Japan) W: Public Health Health-Check Participation Tables (FY2022) [Internet]. 2024,. Available from: https://www.mhlw.go.jp/english/database/db-hh/xlsx/2-16e.xlsx

10. Statistics Bureau of Japan: Population Estimates by Five-Year Age Group, Oct 1, 2023 [Internet]. 2024,. Available from: https://www.stat.go.jp/english/data/jinsui/2023np/index.html

11. Yagi R, Mori Y, Goto S, Iwami T, Inoue K: Routine Electrocardiogram Screening and Cardiovascular Disease Events in Adults. JAMA Intern Med [Internet] American Medical Association, 2024 [cited 2025 Aug 12]; 184:1035–1044. Available from: https://jamanetwork.com/journals/jamainternalmedicine/fullarticle/2820721

12. Saunders NR, Guan J, Fu L, Guo H, Wang X, Guttmann A: Periodic health visits by primary care practice model, a population-based study using health administrative data. BMC Fam Pract [Internet] BioMed Central Ltd., 2019 [cited 2025 Aug 12]; 20:1–8. Available from: https://bmcprimcare.biomedcentral.com/articles/10.1186/s12875-019-0927-6
