# Supplementary material for: Posterior Electrical Axis Deviation Is Associated With Future Development of Left Bundle Branch Block: A UK Biobank Study
Source: JACC Adv. 2026 Jun 17;5(6):102842. doi: 10.1016/j.jacadv.2026.102842 (PMC13308251; doi:10.1016/j.jacadv.2026.102842)
Supplement: Supplemental Permissions for Figure [file mmc2.pdf]

BMJ PUBLISHING GROUP LTD. LICENSE
TERMS AND CONDITIONS

Mar 18, 2026

This Agreement between Mohammad Kayyali - King's College London ("You") and BMJ Publishing Group Ltd. ("BMJ Publishing Group Ltd.") consists of your license details and the terms and conditions provided by BMJ Publishing Group Ltd. and Copyright Clearance Center.

License Number	6231830679832
License date	Mar 18, 2026
Licensed Content Publisher	BMJ Publishing Group Ltd.
Licensed Content Publication	Heart
Licensed Content Title	Histopathological examination of concept of left hemiblock.
Licensed Content Author	J C Demoulin,H E Kulbertus
Licensed Content Date	Aug 1, 1972

Licensed Content Volume	34
Licensed Content Issue	8
Type of Use	Journal/Magazine
Requestor type	Publisher, STM
Reuse sponsored by or associated with a pharmaceutical or medical products company	No
Format	Electronic
Portion	Figure/table/extract
Number of figure/table/extracts	1
Descriptionof figure/table/extracts	Figure 2
Will you be translating?	No
Circulation/distribution	1
Title of new article	Posterior Electrical Axis Deviation is associated with Future Development of Left Bundle Branch Block: A UK Biobank Study

Lead author	Mohammad Kayyali
Title of targeted journal	JACC: Advances
Publisher	Elsevier
Expected publication date	Jun 2026
Portions	Figure 2
The Requesting Person / Organization to Appear on the License	Mohammad Kayyali - King's College London
Requestor Location	Mohammad Kayyali<br>17 Brewhouse Walk<br><br>London, --None-- SE16 6LD<br>United Kingdom
Publisher Tax ID	GB674738491
Payment Type	Invoice
Email Address	mohammad.kayyali@kcl.ac.uk
Billing Address	Mohammad Kayyali<br>17 Brewhouse Walk

	London, United Kingdom SE16 6LD
Total	0.00 GBP

Terms and Conditions

BMJ Terms and Conditions for Permissions

When you submit your order you are subject to the terms and conditions set out below. You will also have agreed to the Copyright Clearance Center's ("CCC") terms and conditions regarding billing and payment https://s100.copyright.com/App/PaymentTermsAndConditions.jsp. CCC are acting as BMJ Publishing Group Limited's ("BMJs") agent.

Subject to the terms set out herein, BMJ hereby grants to you (the Licensee) a non-exclusive, non-transferable licence to re-use material as detailed in your request for this/those purpose(s) only and in accordance with the following conditions:

1) **Scope of Licence:** Use of the Licensed Material(s) is restricted to the ways specified by you during the order process and any additional use(s) outside of those specified in that request, require a further grant of permission.

2) **Acknowledgement:** In all cases, due acknowledgement to the original publication with permission from BMJ should be stated adjacent to the reproduced Licensed Material. The format of such acknowledgement should read as follows:
"Reproduced from [publication title, author(s), volume number, page numbers, copyright notice year] with permission from BMJ Publishing Group Ltd."

3) **Third Party Material**: BMJ acknowledges to the best of its knowledge, it has the rights to licence your reuse of the Licensed Material, subject always to the caveat that images/diagrams, tables and other illustrative material included within, which have a separate copyright notice, are presumed as excluded from the licence. Therefore, you should ensure that the Licensed Material you are requesting is original to BMJ and does not carry the copyright of another entity (as credited in the published version). If the credit line on any part of the material you have requested in any way indicates that it was reprinted or adapted by BMJ with permission from another source, then you should seek permission from that source directly to re-use the Licensed Material, as this is outside of the licence granted herein.

4) **Altering/Modifying Material:** The text of any material for which a licence is granted may not be altered in any way without the prior express permission of BMJ. If adaptation of the material has been approved via bmj.permissions@bmj.com you must include the disclaimer: "Adapted by permission from BMJ Publishing Group Limited. [publication title, author, volume number, page numbers, copyright notice year]

5) **Reservation of Rights:** BMJ reserves all rights not specifically granted in the combination of (i) the licence details provided by you and accepted in the course of this licensing transaction, (ii) these terms and conditions and (iii) CCC's Billing and Payment Terms and Conditions.

6) **Timing of Use:** First use of the Licensed Material must take place within 12 months of the grant of permission.

7) **Creation of Contract and Termination:** Once you have submitted an order via RightsLink and this is received by CCC, and subject to you completing accurate details of your proposed use, this is when a binding contract is in effect and our acceptance occurs. As you are ordering rights from a periodical, to the fullest extent permitted by law, you will have no right to cancel the contract from this point other than for BMJ's material breach or fraudulent misrepresentation or as otherwise permitted under a statutory right. Payment must be made in accordance with CCC's Billing and Payment Terms and conditions. In the event that you breach any material condition of these terms and condition or any of CCC's Billing and Payment Terms and Conditions, the license is automatically terminated upon written notice from BMJ or CCC or as otherwise provided

for in CCC's Billing and Payment Terms and Conditions, where these apply. Continued use of materials where a licence has been terminated, as well as any use of the Licensed Materials beyond the scope of an unrevoked licence, may constitute intellectual property rights infringement and BMJ reserves the right to take any and all action to protect its intellectual property rights in the Licensed Materials.

8) **Warranties:** BMJ makes no express or implied representations or warranties with respect to the Licensed Material and to the fullest extent permitted by law this is provided on an "as is" basis. For the avoidance of doubt BMJ does not warrant that the Licensed Material is accurate or fit for any particular purpose.

9) **Limitation of Liability:** To the fullest extent permitted by law, BMJ disclaims all liability for any indirect, consequential or incidental damages (including without limitation, damages for loss of profits, information or interruption) arising out of the use or inability to use the Licensed Material or the inability to obtain additional rights to use the Licensed Material. To the fullest extent permitted by law, the maximum aggregate liability of BMJ for any claims, costs, proceedings and demands for direct losses caused by BMJ's breaches of its obligations herein shall be limited to twice the amount paid by you to CCC for the licence granted herein.

10) **Indemnity:** You hereby indemnify and hold harmless BMJ and their respective officers, directors, employees and agents, from and against any and all claims, costs, proceeding or demands arising out of your unauthorised use of the Licensed Material.
11) **No Transfer of License:** This licence is personal to you, and may not be assigned or transferred by you without prior written consent from BMJ or its authorised agent(s). BMJ may assign or transfer any of its rights and obligations under this Agreement, upon written notice to you.

12) **No Amendment Except in Writing:** This licence may not be amended except in a writing signed by both parties (or, in the case of BMJ, by CCC on BMJ's behalf).

13) **Objection to Contrary terms:** BMJ hereby objects to any terms contained in any purchase order, acknowledgment, check endorsement or other writing prepared by you, which terms are inconsistent with these terms and conditions or CCC's Billing and Payment Terms and Conditions. These terms and conditions, together with CCC's Billing and Payment Terms and Conditions (which to the extent they are consistent are incorporated herein), comprise the entire agreement between you and BMJ (and CCC) and the Licensee concerning this licensing transaction. In the event of any conflict between your obligations established by these terms and conditions and those established by CCC's Billing and Payment Terms and Conditions, these terms and conditions shall control.

14) **Revocation:** BMJ or CCC may, within 30 days of issuance of this licence, deny the permissions described in this licence at their sole discretion, for any reason or no reason, with a full refund payable to you should you have not been able to exercise your rights in full. Notice of such denial will be made using the contact information provided by you. Failure to receive such notice from BMJ or CCC will not, to the fullest extent permitted by law alter or invalidate the denial. For the fullest extent permitted by law in no event will BMJ or CCC be responsible or liable for any costs, expenses or damage incurred by you as a result of a denial of your permission request, other than a refund of the amount(s) paid by you to BMJ and/or CCC for denied permissions.

15) **Restrictions to the license**:

15.1) **Promotion:** BMJ will not give permission to reproduce in full or in part any Licensed Material for use in the promotion of the following:

a) non-medical products that are harmful or potentially harmful to health

b) medical products that do not have a product license granted by the Medicines and Healthcare products Regulatory Agency (MHRA) or its international equivalents. Marketing of the product may start only after data sheets have been released to members of the medical profession and must conform to the marketing authorization contained in the product license.

16) **Translation**: This permission is granted for non-exclusive world English language rights only unless explicitly stated in your licence. If translation rights are granted, a professional translator should be employed and it must be a true reproduction, accurately conveying the original meaning and of the same quality.

17) **STM Permissions Guidelines**: For content reuse in journals that qualify for permission under the STM Permissions Guidelines (which may be updated from time to time) the terms and conditions of the Guidelines supersede those in this licence.
https://www.stm-assoc.org/intellectual-property/permissions/permissions-guidelines/

18) **General:** Neither party shall be liable for failure, default or delay in performing its obligations under this Licence, caused by a Force Majeure event which shall include any act of God, war, or threatened war, act or threatened act of terrorism, riot, strike, lockout, individual action, fire, flood, drought, tempest or other event beyond the reasonable control of either party.

18.1) In the event that any provision of this Agreement is held to be invalid, the remainder of the provisions shall continue in full force and effect.

18.2) There shall be no right whatsoever for any third party to enforce the terms and conditions of this Agreement. The Parties hereby expressly wish to exclude the operation of the Contracts (Rights of Third Parties) Act 1999 and any other legislation which has this effect and is binding on this agreement.

18.3) To the fullest extent permitted by law, this Licence will be governed by the laws of England and shall be governed and construed in accordance with the laws of England. Any action arising out of or relating to this agreement shall be brought in courts situated in England save where it is necessary for BMJ for enforcement to bring proceedings to bring an action in an alternative jurisdiction.

Other Terms and Conditions:

V1.1

Questions? customercare@copyright.com.
